# Benzomorphan and non-benzomorphan agonists differentially alter sigma-1 receptor quaternary structure, as does types of cellular stress

**DOI:** 10.1007/s00018-023-05023-z

**Published:** 2024-01-09

**Authors:** Simon Couly, Yuko Yasui, Semnyonga Foncham, Ioannis Grammatikakis, Ashish Lal, Lei Shi, Tsung-Ping Su

**Affiliations:** 1 Cellular Pathobiology Section, Integrative Neuroscience Research Branch, Intramural Research Program, National Institute On Drug Abuse, NIH/DHHS, 333 Cassell Drive, Baltimore, MD 21224 USA; 2 Regulatory RNAs and Cancer Section, Genetics Branch, Center for Cancer Research (CCR), National Cancer Institute (NCI), Bethesda, MD 20892 USA; 3 Computational Chemistry and Molecular Biophysics Section, Molecular Targets and Medications Discovery Branch, National Institute On Drug Abuse, NIH/DHHS, 333 Cassell Drive, Baltimore, MD 21224 USA

**Keywords:** Sigma-1 receptor, Endoplasmic reticulum, Mitochondria, MAM, Neurodegenerative disorders

## Abstract

Sigma-1 receptor (S1R) is a calcium-sensitive, ligand-operated receptor chaperone present on the endoplasmic reticulum (ER) membrane. S1R plays an important role in ER-mitochondrial inter-organelle calcium signaling and cell survival. S1R and its agonists confer resilience against various neurodegenerative diseases; however, the molecular mechanism of S1R is not yet fully understood. At resting state, S1R is either in a monomeric or oligomeric state but the ratio of these concentrations seems to change upon activation of S1R. S1R is activated by either cellular stress, such as ER-calcium depletion, or ligands. While the effect of ligands on S1R quaternary structure remains unclear, the effect of cellular stress has not been studied. In this study we utilize cellular and an *in-vivo* model to study changes in quaternary structure of S1R upon activation. We incubated cells with cellular stressors (H_2_O_2_ and thapsigargin) or exogenous ligands, then quantified monomeric and oligomeric forms. We observed that benzomorphan-based S1R agonists induce monomerization of S1R and decrease oligomerization, which was confirmed in the liver tissue of mice injected with (+)-Pentazocine. Antagonists block this effect but do not induce any changes when used alone. Oxidative stress (H_2_O_2_) increases the monomeric/oligomeric S1R ratio whereas ER calcium depletion (thapsigargin) has no effect. We also analyzed the oligomerization ability of various truncated S1R fragments and identified the fragments favorizing oligomerization. In this publication we demonstrate that quaternary structural changes differ according to the mechanism of S1R activation. Therefore, we offer a novel perspective on S1R activation as a nuanced phenomenon dependent on the type of stimulus.

**Supplementary Information:**

The online version contains supplementary material available at 10.1007/s00018-023-05023-z.

## Introduction

S1R is a receptor chaperone protein mainly located at an endoplasmic reticulum (ER) domain that interfaces with the mitochondria, also known as the mitochondria associated endoplasmic reticulum membrane (MAM) [1, 2]. S1R is a ubiquitous protein expressed at different levels across mammalian tissues, with especially high expression in the liver [2–5]. Moreover, S1R expression also varies among different cell types within a tissue. In the brain for example, S1R expression in glial cells differs from that of neurons [6]. Upon activation S1R plays roles in different cellular pathways [2, 7–12]. We observe that these various roles fall under three categories of S1R functions. The most well-known S1R function is its ability to act as a chaperone and stabilize a wide range of proteins [2, 7, 9, 13–15]. For example, S1R stabilizes Ip3R3, which exports calcium from the ER into the mitochondria [2], and IRE1, an ER transmembrane protein necessary to the proper activation of the unfolded protein response (UPR) [7, 8]. S1R has also been observed to bind cholesterol and remodel lipid rafts on the ER membrane [10, 16, 17]. More recently, S1R has been found to interact with RNA and more specifically G4C2 [14, 18].

The downstream effects of S1R activation with exogenous ligands have been studied previously [2, 18–20]; however, the endogenous activation of S1R is less understood. Neurosteroids are the main endogenous ligands suspect. Progesterone is known to act as an antagonist while pregnelonone shows some agonist properties [21, 22]. Furthermore, S1R has been shown also to be activated following cellular stress such as ER calcium depletion and oxidative stress [2, 12, 23]. The structure of S1R has been recently elucidated by important discoveries [24–26]. Crystallography analysis shows S1R as a single transmembrane protein with a ligand binding pocket on the C-terminal [25]. Moreover the transmembrane domain, located on the N-terminal, seems also to contain a cholesterol-binding motif [17]. Despite recent publications demonstrating that the structure of S1R could affect its antiviral function [27] or its ability to shape ER membrane [28], the relationship between S1R structure and function is not yet totally clear. At resting state S1R is found as monomers and homomers [29], and upon activation S1R seems to tend toward monomeric conformation [30]. However, these previously published results seem to be dependent on the position of the tags used to observe the protein structures [31]. Moreover, these experiments rely on the use of exogenous ligands; the consequences of cellular stress such as ER-calcium depletion and oxidative stress on the quaternary structure of S1R remain unidentified. In this study we develop models in order to shed the light on the effect of exogenous ligands on S1R structure, but also, we show for the first time the consequences of cellular stress on the oligomerization of S1R. This paper provides more evidence of the importance of S1R C-terminal for the oligomerization process.

## Materials and methods

### Cell culture

Neuro2a (N2a) cell line was purchased from American Type Cell Collection (CCL-131). Cells were maintained and grown in complete culture Dulbecco’s modified Eagle’s medium (DMEM; GIBCO, 11,965–092) containing 10% fetal bovine serum (FBS) (R&D systems #S11150) and 1% penicillin–streptomycin (GIBCO, 15,140–122). Cells were passaged every 2–3 days and maintained at 37 °C in a 5% CO_2_ incubator.

### Production of a N2a cell line expressing endogenous Flag-tagged S1R

To tag C-terminal of S1R with a Flag peptide, we utilized prime editing, specifically the prime editor 3 system, with an engineered prime editing guide RNA (epegRNA) and followed the protocols described in Nelson et al. and Anzalone et al. [32, 33]. Necessary plasmids were obtained through addgene: pCMV-PE2-P2A-GFP and pU6-tevopreq1-GG-acceptor were a gift from David Liu (Addgene plasmid # 174038) [32]* .*

(Addgene plasmid # 132776) [33] and BPK1520 was a gift from Keith Joung (Addgene plasmid # 65777) [34].

For selecting sequences of epegRNA and nicking single guide RNA (ngRNA), we used PrimeDesign [35] and pegFinder [36]. The following oligos were used to assemble epegRNA and cloned into pU6-tevopreq1-GG-acceptor: protospacer oligos (top: CACCGTCCTTCAGGCCTGGCTGGTCGTTTT, bottom: CTCTAAAACGACCAGCCAGGCCTGAAGGAC), 3’-extension oligos (top: GTGCTTGAGCTTACCACCTACCTCTTTGGCCAAGACTCCGACTACAAAGACGATGACGACAAGTGACCAGCCAGGCCCATATATA, bottom: CGCGTATATATGGGCCTGGCTGGTCACTTGTCGTCATCGTCTTTGTAGTCGGAGTCTTGGCCAAAGAGGTAGGTGGTAAGCTCAA) and scaffold oligos (top: AGAGCTAGAAATAGCAAGTTAAAATAAGGCTAGTCCGTTATCAACTTGAAAAAGTGGCACCGAGTCG, bottom: GCACCGACTCGGTGCCACTTTTTCAAGTTGATAACGGACTAGCCTTATTTTAACTTGCTATTTCTAG). ngRNA oligos (top: CACCGATGACGACAAGTGACCAGCC, bottom: AAACGGCTGGTCACTTGTCGTCATC) were cloned into BPK1520.

Wild type (Wt) N2a cells were seeded at 1.5 × 10^5^ cells per well in a 12-well plate. In the following day, the cells were transfected with plasmid DNAs (3.33ug of pCMV-PE2-P2A-GFP, 833 ng of epegRNA plasmid, and 333 ng of ngRNA plasmid) using lipofectamine 2000 (Invitrogen). The plasmids and 6 μl of lipofectamine 2000 were separately diluted in 100 μl each of Opti-MEM (Gibco), and then the solutions were combined and added to the cells in 1 ml of fresh growth medium. After 48 h of incubation, cells were trypsinized and single cells expressing moderate level of GFP were sorted in 96-well plates using BD FACSAria II cell sorter (BD Biosciences). The 96-well plates contained growth medium prepared from 50% fresh medium and 50% used medium which was collected from culture dish of N2a cells and filtered. After single cells grew, cells from 48-well plates were collected and incubated in 50–100 μl of proteinase K lysis buffer [50 mM Tris–HCl pH8.0, 1 mM EDTA pH8.0, 0.5% Tween-20 and 1 mg/ml proteinase K from Tritirachium album (Boehringer Mannheim)] overnight at 50 °C. Proteinase K was inactivated by incubating at 95 °C for 10 min, and then the resulting genome solution was used as a template for PCR. PCR was performed to amplify a fragment containing the C-terminus of S1R using Taq DNA polymerase with standard Taq buffer (NEB), forward primer: TCATTCCCCTCCTCTCCATA and reverse primer: CAGCAGGAGGCTCTAGGAAA. The PCR product was purified with High Pure PCR product Purification kit and sent for sanger sequencing (Azenta Life Science) with the reverse primer mentioned above. At first, we obtained heterozygous (Flag-tag ±) so we repeated the procedure using the heterozygous cells to obtain homozygous (Flag-tag + / +).

### Generation of S1R-knockout (KO) N2a cell line

Two of the pSpCas9 BB-2A-Puro (PX459) plasmids containing CRISPR guide RNA (gRNA) sequence targeting the mouse Sigma-1 receptor were obtained from GenScript (SC1948-459). The gRNA sequences, 5ʹ-GGCCCCGGGCATAGGCCCGA-3ʹ and 5ʹ-CGCTAGAATGCCGTGGGCCG-3ʹ were used. Two plasmids were mixed in equal amount and transfected into N2a cells. At 48 h after transfection, cells were treated with 2 µg/ml puromycin for 7 days. Cells were trypsinized and resuspended to a density of 8–10 cells/ml and 100 µL each of the cell suspension was transferred to a well of a 96-well plate. Expanded cells were collected and cell lysates were analyzed for the S1R receptor protein expression by western blot by using the Santa Cruz Biotechnology B5 anti-S1R receptor antibody.

### Transfection

Cell monolayers of 70% density in 6 wells-plate were used for transfection with plasmids using PolyJet reagent (SignaGen Laboratories, SL100688). In all, the PolyJet reagent and plasmids ratio (2:1) were incubated in 0.2 mL serum-free DMEM for 12 min at room temperature. Subsequently, mixed DNA-polyJet complexes were added into each well. Media was refresh after overnight incubation. Cells were then incubated at 37 °C in a 5% CO_2_ incubator (Thermo Fisher Scientific) for 48 h before drug application and protein extraction.

### Drug preparation and application

Drugs were freshly prepared the day of the experiment. For cellular experiments drugs were resuspended in Dulbecco’s modified Eagle’s medium (DMEM; GIBCO, 11,965–092) containing 10% FBS and 1% penicillin–streptomycin (GIBCO, 15,140–122) at concentrated concentration to reach final concentration mentioned in each figure. Before drug application media was refreshed and drugs were administered directly in the final volume. (1R,9R,13R)-1,13-dimethyl-10-(3-methylbut-2-enyl)-10-azatricyclo [7.3.1.02,7] trideca-2(7),3,5-trien-4-ol (( +)-Pentazocine) was from the NIDA IRP (Baltimore, MD, USA); N, N-Dipropyl-2-(4-methoxy-3-(2-phenylethoxy) phenyl) ethylamine monohydrochloride (NE100) from Tocris 3133; 1-[2-(3,4-Dichlorophenyl) ethyl]-4-methylpiperazine (BD1063) from Tocris (0883); ( ±)-1,2,3,4,5,6-hexahydro-6,11-dimethyl-3-(2-propen-1-yl)-2,6-methano-3-benzazocin-8-ol ((+)-SKF10047) from NEN Life Science Products; 2-morpholin-4-ylethyl 1-phenylcyclohexane-1-carboxylate (PRE-084) Tocris (0589); methyl(1R,2R,3S,5S)-3-benzoyloxy-8-methyl-8-azabicyclo[3.2.1]octane-2-carboxylate;hydrochloride (HCl-cocaine) was from the NIDA IRP (Baltimore, MD, USA).

### Protein extraction

For animal tissues. After sacrificing the animal, liver was quickly extracted and immersed in radioimmunoprecipitation assay (RIPA) buffer (50 mM Tris pH7.4, 150 mM NaCl, SDS 0.1%, 1% Triton X100) supplemented with EDTA-free protease inhibitor cocktail tablets (Complete, Mini, EDTA-free; Roche Diagnostics, 11,836,170,001) and phosphatase inhibitor cocktail tablets (PhosStop, Millipore, PHOSS-RO). For 10 mg of liver 600 µL of buffer was used. Samples were homogenized with glass homogenizer and incubated 5 min on ice. Then samples were centrifuged for 3 min at 1000 g at 4 °C and the supernatant was kept for western blot analysis.

For cells. First cells were washed with 1 × phosphate buffered saline (PBS, gibco) and harvested by detaching them with a scraper and collected in 1.5 mL tubes. Then tubes were centrifuged at 600 g for 3 min and pellet were lysed. For oligomers/monomers analysis cell were lysed with 100 µL of modified RIPA buffer (50 mM Tris–HCl, pH 7.4, 150 mM NaCl, 0.05% sodium dodecyl sulfate (SDS), 0.5% Triton X-100, and 0.05% sodium deoxycholate [SigmaAldrich, D6750]) supplemented with EDTA-free protease inhibitor cocktail tablets (Complete, Mini, EDTA-free; Roche Diagnostics, 11,836,170,001) and phosphatase inhibitor cocktail tablets (PhosStop, Millipore, PHOSS-RO). Then cells were incubated on ice for 10–15 min. Eventually each tube were centrifuged 10 min at 18,000 *g* 4 °C and supernatant collected and stored at − 80 °C for latter protein analysis.

### Western blot

Protein concentrations of cell lysates were determined using BCA protein assay kit (Thermo Fisher Scientific, 23,225). Laemmli 4X sample buffer (Bio-Rad, 161–0747) containing 10% 2-mercaptoethanol was added to 30 µg of protein. Then samples were heated for 5 min at 90 °C, except during experiment aiming to measure the ratio monomer/oligomers. These protein samples were separated by using 0.1% SDS–polyacrylamide gel electrophoresis (SDS-PAGE) containing 2% of 2,2,2-Trichloroethanol to visualize the quantity of total protein in each lane and transferred overnight at 4 °C and 30 mA onto a polyvinylidene difluoride membrane. After incubation with 2% BSA in TBST (Tris-buffered saline with 0.1% Tween 20 [Bio-Rad Laboratories, 170–6531]) for 1 h, membranes were incubated with primary antibody anti-S1R (Polyclonal antibody, 15168-1-AP, 1:1000) for an 1 h at room temperature. Membranes were washed 3 times with TBST for 10 min followed by secondary antibody anti-rabbit IgG 680 (Licor, 926-68071, 1:10,000) for 1 h at room temperature. Blots were washed 3 times for 10 min with TBST and developed by using the Li-Cor odyssey CLx and band intensity was analyzed by Image Studio Lite (LiCor 5.2.5) according to the manufacturer’s manual.

### Co-immunoprecipitation

N2a cells in 6-well plates were transfected with 2 μg of rat S1R-V5 construct. After 48 h cells were washed with 1 mL 1X PBS (GIBCO, 20012-027) and harvested with 500 µL of PBS. lysed using CHAPS lysis buffer (50 mM Tris pH 7.4, 150 mM NaCl, 0.5% CHAPS and supplemented with EDTA-free protease inhibitor cocktail tablets (Complete, Mini, EDTA-free; Roche Diagnostics, 11,836,170,001) on ice for 30 min. Protein concentrations of cell lysates were determined using BCA protein assay kit (Thermo Fisher Scientific). one μg of anti-Flag rabbit antibody (Proteintech, 80010), or rabbit normal IgG added with 5 μL of Dynabeads Protein G (Invitrogen, 100003D) were mixed and incubated at room temperature for 15 min. Three hundred μg of cell lysate was added to the antibody-beads mixture and mixed overnight at 4 °C. Beads were washed with 500 μL of CHAPS lysis buffer three times, then beads were resuspended in 30 μL of 2 × Laemmli sample buffer (Bio-Rad) and heated at 70 °C for 15 min. These protein samples were separated by 11% SDS-PAGE and transferred onto a polyvinylidene difluoride membrane. After incubation with 2% BSA in TBST for 1 h, membranes were incubated with anti-V5 antibody (Invitrogen, R960-25, 1:250) for 1 h at room temperature. Membranes were washed 3 times with TBST for 10 min followed by secondary antibody anti-mouse IgG 800 (Licor, 926-32210, 1:10,000) for 1 h at room temperature. Blots were washed 3 times for 10 min with TBST and developed by using the Li-Cor odyssey CLx. Then, membranes were incubated overnight with anti-Flag antibody (Proteintech, F3165, 1:1000) at 4 °C, washed with TBST, and incubated with anti-mouse IgG 800 for 1 h at room temperature. Immunoreactive proteins were visualized using Li-Cor odyssey CLx, band intensity was analyzed by Image Studio Lite (LiCor 5.2.5) according to the manufacturer’s manual.

### Animals

All methods and animal procedures were conducted in accordance with the principles as indicated by the NIH Guide for the Care and Use of Laboratory Animals. These animal protocols were also reviewed and approved by the NIDA intramural research program Animal Care and Use Committee, National Institute of Health. S1R KO mice were generated as previously described [37]. We obtained the mice of Oprs1 mutant (+ / −) OprsGt(IRESBetageo)33Lex litters on a C57BL/6 J × 129 s/SvEv mixed background from the Mutant Mouse Regional Resource Center of the University of California, Davis. The S1R (+ / −) males were backcrossed for 10 generations to females on C57BL/6 J and then, mice were further genotyped. Mice were maintained in a 12 h day/night cycle facility with free access to food and water. In rodent experiments, drugs were solubilized in physiological saline (vehicle solution) and administered intraperitoneally (IP) at the dose of 10 mg/Kg of ( +)-Pentazocine, at 5 mL/kg body weight for 30 min.

### Software

All statistical analysis and graphics were made with Prism 9 for macOS. Graphical summary and artwork made with Adobe Illustrator 2023, Microsoft PowerPoint and ChemDraw 21.0.0 for Mac.

## Results

### (+)-Pentazocine induces monomerization of S1R in N2a cells and mice model

**Fig. 1:**
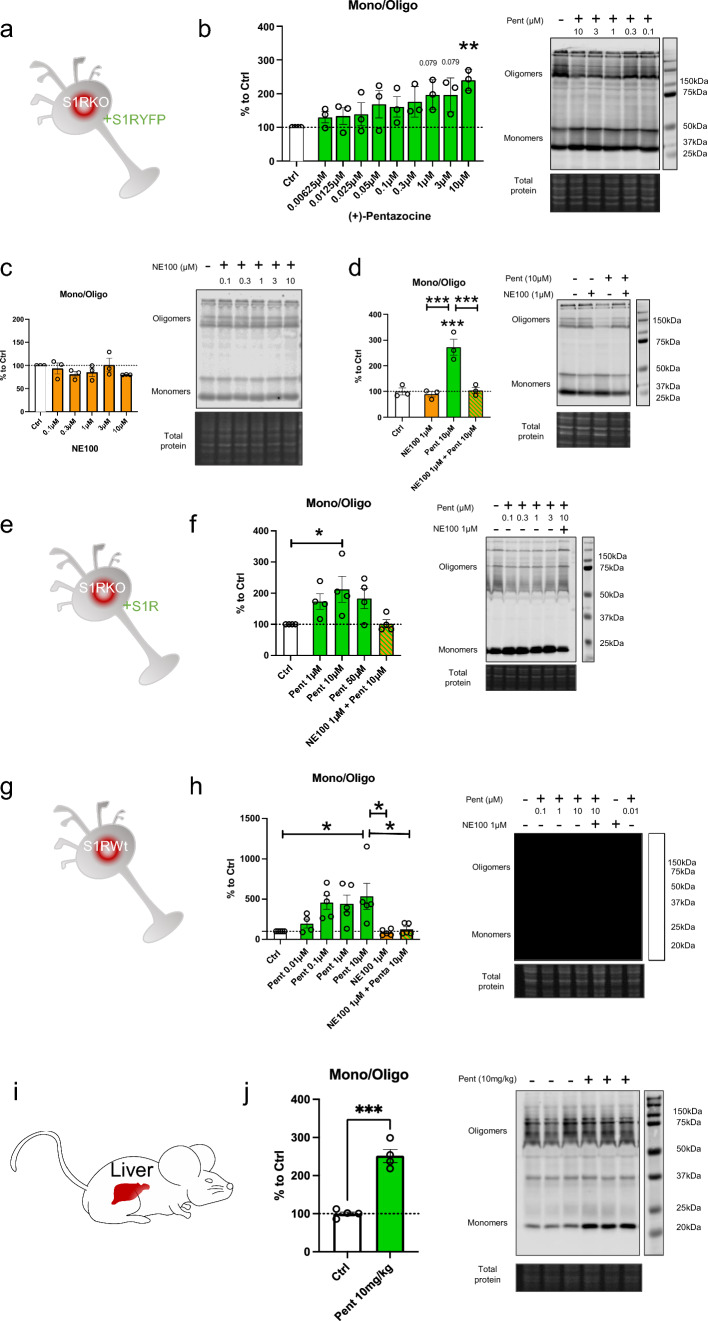
(+)-Pentazocine induces monomerization of S1R in N2a cells, which is blocked by pre-incubation of NE-100. **a** Representation of the model used: Overexpression of S1R tagged on the C-terminal with YFP in S1R KO N2a cells. **b **left Bar graph represents immunoblot analysis of the ratio of S1R monomers’ signal intensity to oligomers’ signal intensity (ratio mono/oligo) in the control condition or with incubation of ( +)-Pentazocine for 30 min at different concentrations (0.00625 to 10 µM). A 10 µM ( +)-Pentazocine incubation induces a significant increase in the ratio mono/oligo. **b** right Representative immunoblot showing oligomeric and monomeric forms of the S1R-YFP. Total protein was used as loading control. **c** left Bar graph represents immunoblot analysis of the ratio mono/oligo with incubation of NE100 for 30 min at different concentration (0.1 to 10 µM). No effect of the NE100 incubation is observed. **c** right Representative immunoblot showing S1R-YFP oligomers and monomers after incubation with NE100. **d** left Bar graph represents immunoblot analysis of incubation with 1 µM of NE100 for 35 min, incubation of ( +)-Pentazocine and co-incubation of NE100 (1 µM) and ( +)-Pentazocine (10 µM) for 30 min preceded by pre-incubation of NE100 alone (1 µM) for 5 min. The NE100 pre-incubation blocks the effect of ( +)-Pentazocine on the ratio mono/oligo. **d **right Representative immunoblot showing S1R-YFP oligomers and monomers after incubation with NE100 and/or ( +)-Pentazocine. **e** Representation of the model used: Overexpression of S1R in S1R KO N2a cells. **f** left Bar graph represents immunoblot analysis of the impact of ( +)-Pentazocine (1 to 50 µM) incubation with or without the pre- and co-incubation of NE100 (1 µM) on the ratio mono/oligo. A 10 µM ( +)-Pentazocine incubation induces an increase in the ratio mono/oligo, which is blocked by the pre-incubation of NE100 (1 µM). **f** right Representative immunoblot showing S1R oligomers and monomers. **g** Representation of the model used: Endogenous S1R in Wt N2a cells. **h** left Bar graph represents immunoblot analysis of the impact of ( +)-Pentazocine (0.01 to 10 µM) incubation with or without the pre- and co-incubation of NE100 (1 µM) on the ratio mono/oligo. A 10 µM ( +)-Pentazocine incubation induces a significant increase in the ratio mono/oligo, which is blocked by the pre- and co-incubation of NE100 (1 µM). **h** right Representative immunoblot showing endogenous S1R oligomers and monomers. **i** Representation of the model used: Endogenous S1R in liver extract from Wt Mice.** j** left Bar graph represents immunoblot analysis of the impact of IP injection of ( +)-Pentazocine (10 mg/kg) on the ratio mono/oligo. A 10 µM ( +)-Pentazocine incubation induces a significant increase in the ratio mono/oligo. **j** right Representative immunoblot showing endogenous S1R oligomers and monomers. Significant differences were found using t test or ordinary one-way ANOVA followed by a Tukey’s multiple comparisons test (*p < 0.05; **p < 0.01, ***p < 0.001) or unpaired t test (***p < 0.001)

It has been previously shown that S1R agonists are able to induce change in the quaternary structure of S1R [30, 31, 38]. However, most of these studies use tagged S1R and it has been demonstrated that the position of the tag (N-terminal or C-terminal) could impact the effect of the agonist on the structure [31, 39]. Moreover, these analyses are mainly based on S1R overexpression experiments which could also impact the physiological behavior of the protein. In this study we started by determining how ( +)-Pentazocine impacts overexpressed C-terminal tagged and untagged S1R in S1R KO N2a cells, and endogenous S1R in Wt N2a cells and in Wt mice liver (Fig. 1). To do so we processed protein extracts without thermal denaturation. This allowed us to observe different sizes of S1R (Supplementary Information 1). The overexpression of untagged S1R yielded monomers around 24 kDa and oligomers at 75 kDa and 150 kDa (Supplementary Information 1a), while YFP-tagged S1R yielded monomers around 50 kDa, fragments at 37 kDa, and oligomers around 100 kDa and 200 kDa (Supplementary Information 1b). Meanwhile, S1R KO samples do not provide any signal under either protein processing condition. It has to be noted that in order to have a clearer understanding of the oligomerization results we conducted the S1R overexpression experiments in S1R KO condition, so that we only measured signals coming from one source of S1R. Using this method, we first observed in S1R KO N2a cells overexpressing S1R-YFP (Fig. 1a) that 30 min incubation with (+)-Pentazocine at 10 µM can increase S1R monomer concentration at the detriment of S1R oligomer concentration (Fig. 1b). We then observed the effect of 30 min incubation with NE100, a S1R antagonist. We noted no effect of NE100 by itself at different concentrations between 0.1 and 10 µM (Fig. 1c). Finally, we pre-incubated NE100 for 5 min before applying (+)-Pentazocine at 10 µM for 30 min. In this condition we noted a blockade of ( +)-Pentazocine ability to induce S1R monomerization (Fig. 1d). Then to see the potential impact of the tag we analyzed the effect of ( +)-Pentazocine incubation (with or without pre-incubation with NE100 1 µM) in S1R KO N2a cells expressing untagged S1R (Fig. 1e). We observed that ( +)-Pentazocine, in this context also, increases the monomerization of S1R at the detriment of oligomerization. This is also blocked by the pre-incubation of NE100 (Fig. 1f). Although the overexpression of S1R increases the quality of the Western Blot signal, it could also alter the behavior of S1R oligomerization compared to a physiological concentration of S1R. Therefore, we measured the ratio of monomers/oligomers of endogenous S1R in Wt N2a cells (Fig. 1g). We noted that ( +)-Pentazocine is also able in this condition to induce monomerization of S1R and this is inhibited by the pre-incubation of NE100 (Fig. 1h). Interestingly in Wt condition we observe a larger shift from oligomers to monomers compared to overexpression with the same ( +)-Pentazocine concentration. This could be due to the quantity of S1R available in the cells, which is higher in overexpressed conditions compared to conditions with endogenous S1R, where a lower quantity of S1R is available for a same quantity of ligands. In mammals, the liver is one of the organs that express S1R at the highest level [2]. In order to confirm what was described earlier *in-vivo,* we performed the protein analysis using liver tissue from Wt mice at 30 min after the intraperitoneal injection of saline or ( +)-Pentazocine at 10 mg/kg (Fig. 1i). We noted here also a drastic increase of the S1R monomers/oligomers ratio after the injection of ( +)-Pentazocine (Fig. 1j). At resting state, the concentration of oligomers is higher than the concentration of monomers both in cells and liver (Supplementary information 1c). When compared the monomer/oligomer ratios of cells and liver, liver seems to have higher ratio than cells (Supplementary information 1d), however these results may be affected by different experimental conditions such as sample preparation and transfer time. Altogether, these experiments strengthened previous results demonstrating that S1R agonist increases monomerization of S1R. Furthermore, our results show that neither C-terminal tags nor overexpression changes the impact of S1R agonist on S1R quaternary structure when compared to conditions containing only endogenous S1R. This result has previously been observed only *in-vitro*, and for the first time this result is shown to occur *in-vivo*.

### Co-immunoprecipitation of S1Rs is decreased by the in-vitro incubation of (+)-Pentazocine, and this is inhibited by the pre-incubation of NE100

**Fig. 2:**
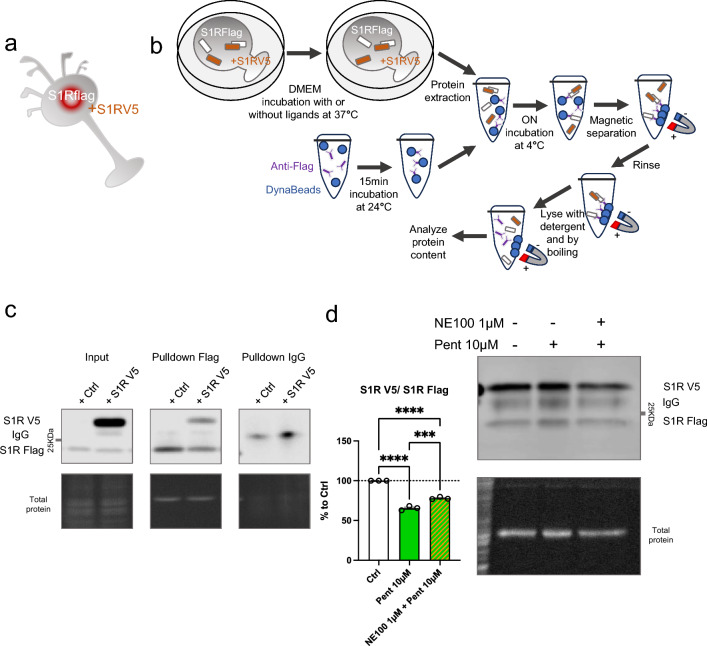
Co-immunoprecipitation of S1R decreased by the incubation of ( +)-Pentazocine and it is blocked by the preincubation with NE100.** a** Representation of the model used: Overexpression of S1R tagged with V5 in N2a cells expressing endogenous S1R tagged with Flag. **b** Representation of the experiment: cells were incubated for 30 min with media (Ctrl), media containing ( +)-Pentazocine (10 µM), or media containing ( +)-Pentazocine and NE100 (1 µM). Then protein was extracted with modified RIPA buffer. Anti-Flag antibodies were incubated with DynaBeads. Protein extract and Anti-Flag/Dynabeads complex were incubated overnight together to allow the binding of Anti-Flag/Dynabeads complex with S1R-Flag. After several washes, the pull-downed proteins were analyzed with Western blot. **c** Immunoblot showing protein content from overexpressing S1R-V5 (S1R V5) or non-transfected cells (Ctrl). Membrane was incubated with mouse antibody targeting V5 and mouse antibody targeting Flag **c** left Immunoblot shows the protein extract input before immunoprecipitation. **c** middle Immunoblot shows the protein content after immunoprecipitation with rabbit antibody targeting Flag. **c** right Immunoblot shows the protein content after immunoprecipitation with IgG rabbit. **d** Bar graph represents immunoblot analysis of the S1R. Significant differences were found using ordinary one-way ANOVA followed by a Tukey’s multiple comparisons test (***p < 0.001, ****p < 0.0001)

To confirm the finding that monomer concentration increases, and oligomer concentration decreases upon application of S1R agonist, we used co-immunoprecipitation of S1Rs (Fig. 2). Since it has been challenging to pull down endogenous S1R using immunoprecipitation, we created N2a cells expressing S1R endogenously Flag-tagged in the C-terminus using prime editing technology [32, 33, 40] (Supplementary Information 2). First, we transfected these cells with S1R with a C-terminal V5 tag (Fig. 2a). Two days after the transfection we incubated the cells with regular media (control) or media containing ( +)-Pentazocine (10 µM), with or without pre-incubation of NE100 (1 µM). We then performed the protein extraction followed by the co-immunoprecipitation. The immunoprecipitation was conducted with magnetic beads coated with anti-Flag antibodies as shown in Fig. 2b. Pull-downed proteins were then analyzed with western blot. Membranes were blotted with anti-Flag and anti-V5 antibodies (Fig. 2c). The signal intensities of co-immunoprecipitated S1R-V5 and S1R-Flag were then measured and the ratio of S1R-V5/S1R-Flag was analyzed (Fig. 2d). We noted that the quantity of co-immunoprecipitated S1R-V5 decreased with the incubation of ( +)-Pentazocine compared to that of the control. Interestingly we were able to inhibit this decrease by pre-incubating with NE100 before applying ( +)-Pentazocine. These results confirm the previous observations seen in Fig. 1 and support the hypothesis that agonists can increase monomer concentration and decrease oligomer concentration and that this is blocked by the pre-incubation of antagonists.

### Benzomorphan-based agonists act on the quaternary structure of S1R, while non-benzomorphan-based agonists do not

**Fig. 3:**
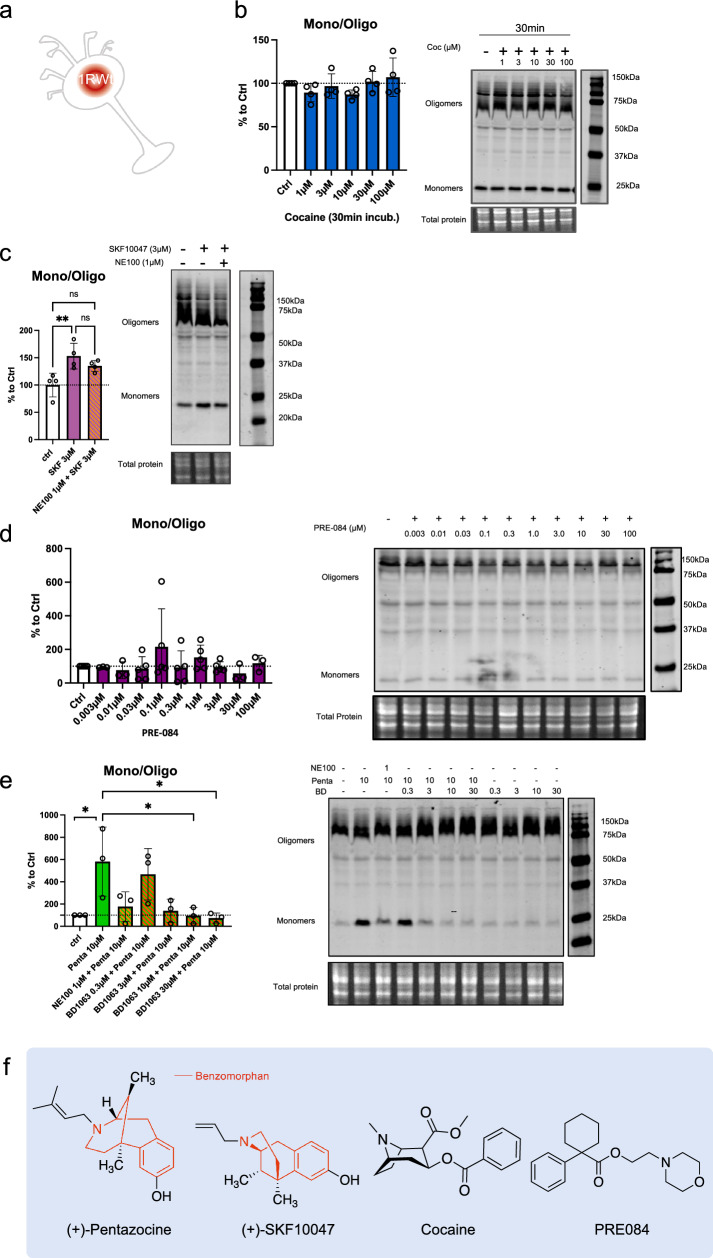
Cocaine and PRE-084 do not induce monomerization while ( +)-SKF10047 does. BD1063, as NE100, is able to block ( +)-Pentazocine effect.** a **Representation of the model used: Endogenous S1R in Wt N2a cells. **b** left Bar graph represents immunoblot analysis of the S1R ratio of mono/oligo in control condition or with incubation of cocaine for 30 min at different concentrations (1 to 100 µM). None of the dosages affect S1R quaternary structure. **b** right Representative immunoblot showing S1R oligomers and monomers after incubation with cocaine. **c** left Bar graph represents immunoblot analysis of incubation of ( +)-SKF10047 (3 µM) for 30 min with or without pre-incubation of NE100 (1 µM) for 5 min. ( +)-SKF10047 (3 µM) induces monomerization of S1R. NE100 pre-incubation seems to block the effect of ( +)-SKF10047 (3 µM) on the ratio mono/oligo. **c** right Representative immunoblot showing S1R oligomers and monomers after incubation of ( +)-SKF10047 with or without pre-incubation with NE100 (1 µM). **d** left Bar graph represents immunoblot analysis testing the effect of PRE-084 (0.003 to 100 µM) incubation for 30 min on the ratio mono/oligo. No effect of PRE-084 incubation is observed. d right Representative immunoblot showing S1R oligomers and monomers after incubation with PRE-084. e left Bar graph represents immunoblot analysis of the ratio of S1R monomers’ signal intensity over oligomers’ signal intensity (ratio mono/oligo) in control condition or with the 30 min incubation of ( +)-Pentazocine (10 µM), ( +)-Pentazocine (10 µM) with pre incubation of NE100 (1 µM) and ( +)-Pentazocine (10 µM) with pre-incubation of BD1063 at different concentration (0.3 to 30 µM). BD1063 pre-incubation at 10 µM or higher blocks the effect of ( +)-Pentazocine. **e** right Representative immunoblot showing oligomeric and monomeric forms of S1R. **d** ( +)-Pentazocine, ( +)-SKF10047, cocaine, PRE084, molecular structures. Benzomorphan groups are highlighted in red. Significant differences were found using ordinary one-way ANOVA followed by a Tukey’s multiple comparisons test (*p < 0.05, **p < 0.01)

Cocaine is known to bind S1R and is considered to act as an agonist since S1R antagonists are able to mitigate its effect [41–43]. However, as S1R is not a classic receptor with a well define cellular pathway the definition itself of agonist and antagonist could vary. The nuance is even more important when it’s concerning molecules that does not only target S1R and has consequences on cellular metabolisms that does not relay on S1R. Therefore, we sought to determine whether cocaine can affect the quaternary structure of S1R as other more specific ligands can do (Fig. 3). We began by observing the potential agonist ability of cocaine, *i.e.* to induce monomerization (Fig. 3a, b). We incubated S1R Wt N2a cells with different concentrations of cocaine (1, 3, 10, 30, 100 µM) for 30 min before starting protein extraction. We did not observe any effect of cocaine on the S1R monomers/oligomers ratio. We also examined the effect of a 30 min and 24 h incubation of cocaine on S1R KO cells overexpressing YFP tagged S1R but we also found no effect (Supplementary Information 3a, b, c). Next, we measured the ability of cocaine to act as an antagonist (Supplementary Information 3d). We pre-incubated with cocaine (1, 10, 100 µM) for 5 min before applying (+)-Pentazocine (10 µM) which was previously shown to induce monomerization (Fig. 1). We noted that the pre-incubation of cocaine does not block the effect of ( +)-Pentazocine (10 µM) as NE100 was able to (Fig. 1). To explore the effect of other S1R ligands we repeated these experiments on Wt N2a cells with ( +)-SKF10047 and PRE-084, known as S1R agonist, and BD1063, known as S1R antagonist [19, 20, 44]. First, we tested different concentrations of ( +)-SKF10047 (0.03 to 300 µM) on the monomerization of S1R, allowing us to select an effective dose of 3 µM (Supplementary Information 3e, f). We assessed the effect of ( +)-SKF10047 (3 µM) on S1R structure with or without pre-incubation of NE100 (1 µM) (Fig. 3c). We found that ( +)-SKF10047 induce monomerization of S1R as does ( +)-Pentazocine. Furthermore, the pre-incubation of NE100 to reduce the monomerization effect induced by ( +)-SKF10047 as incubation of both, ( +)-SKF10047 and NE100 does not present different ratio compared to our control condition. Next, we measure the effect of PRE-084 on S1R quaternary structure (Fig. 3d). We note that PRE-084, despite trying a broad range of concentrations, does not impact the monomers/oligomers ratio. We also tested different concentrations of pre-incubated BD1063 (0.3, 3, 10, 30 µM) on the effect of ( +)-Pentazocine (10 μM) (Fig. 3e). At concentrations of 3, 10 and 30 µM, BD1063 is able to block the monomerization induced by ( +)-Pentazocine, just like we observed with NE100 (Fig. 1c). Moreover, similar to NE100, BD1063 does not seems to have an effect on quaternary structure when applied alone (Supplementary Information 3 g). Interestingly it has to be noted that both ( +)-Pentazocine and ( +)-SKF10047 structures include a benzomorphan group, whereas cocaine and PRE-084 do not (Fig. 3f). We propose that S1R agonists with benzomorphan group are able to change the quaternary structure of S1R while agonists without benzomorphan group cannot.

### H_2_O_2_ but not thapsigargin induces S1R monomerization

**Fig. 4:**
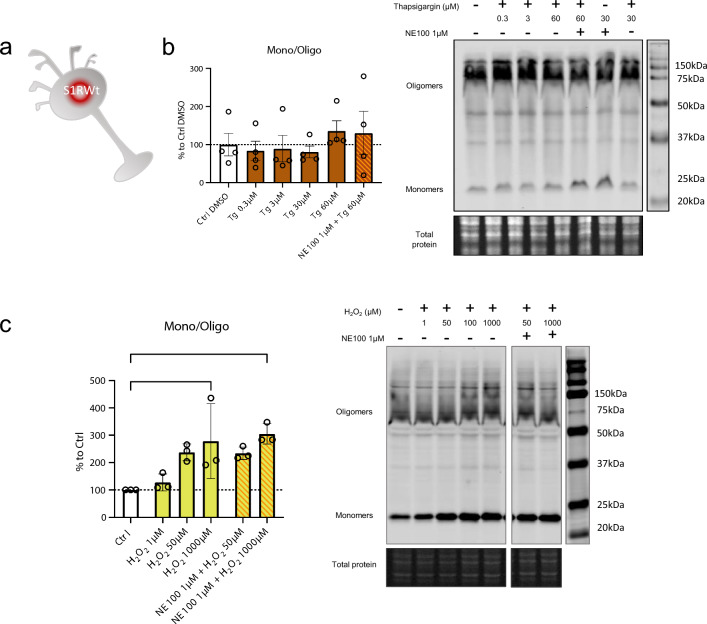
Thapsigargin (Tg) does not impact oligomerization states of S1R meanwhile H_2_O_2_ is. **a** Representation of the model used: Endogenous S1R in Wt N2a cells. b left Bar graph represents immunoblot analysis of the S1R ratio mono/oligo in control condition or with incubation of Thapsigargin for 60 min at different concentrations (0.3 to 60 µM) with or without pre-incubation with NE100 (1 µM). All conditions are incubated with 0.6% DMSO. None of the conditions affect S1R quaternary structure. **b** right Representative immunoblot showing S1R oligomers and monomers after incubation with Thapsigargin and NE100 (1 µM). **c** left Bar graph represents immunoblot analysis of the S1R ratio mono/oligo in control condition or with the incubation of H_2_O_2_ for 30 min at different concentration (1 µM to 1000 µM) with or without pre-incubation with NE100 (1 µM) for 5 min. 1 mM of H_2_O_2_ induces a monomerization of S1R and pre-incubation of NE100 did not block this effect. **c** right Representative immunoblot showing S1R oligomers and monomers after incubation with H_2_O_2_ and NE100 (1 µM). Significant differences were found using ordinary one-way ANOVA followed by a Tukey’s multiple comparisons test (*p < 0.05)

S1R is known to be activated, i.e., to initiate the separation of S1R and BiP, by cellular stress such as ER calcium depletion [2] and oxidative stress [23, 45]. From a molecular point of view, the way that these phenoma impact S1R and its structure is not known. Therefore, we investigate if cellular stress alters S1R quaternary structure. We used Wt N2a cells incubated with either thapsigargin (inducing ER calcium depletion) or H_2_O_2_ (increasing oxidative stress) (Fig. 4a). Interestingly the incubation of thapsigargin at different concentrations (0.3, 3, 30, 60 µM) does not to induce any significant change in the S1R monomers/oligomers ratio (Fig. 4b). However, the application of H_2_O_2_ at 1000 µM significatively increase the monomerization of S1R (Fig. 4c). We observe that the pre-incubation of NE100 before application of H_2_O_2_ does not seem to decrease the effect of H_2_O_2_. We did not find any decrease in protein concentration while processing samples; therefore, the tested concentrations and time of incubation of thapsigargin and H_2_O_2_ did not seem to induce massive cell death (Supplementary Information 4a, b). This suggests that S1R quaternary structural changes occur at sub-toxic oxidative stress levels. These results demonstrate that the quaternary structure of S1R is differentially affected by different activators. Exogenous agonists induce monomerization and are blocked by antagonists. ER calcium depletion does not induce monomerization. Oxidative stress induces monomerization but does not seem to be affected by the presence of an antagonist.

### S1R C-terminus is highly involved in oligomerization

**Fig. 5:**
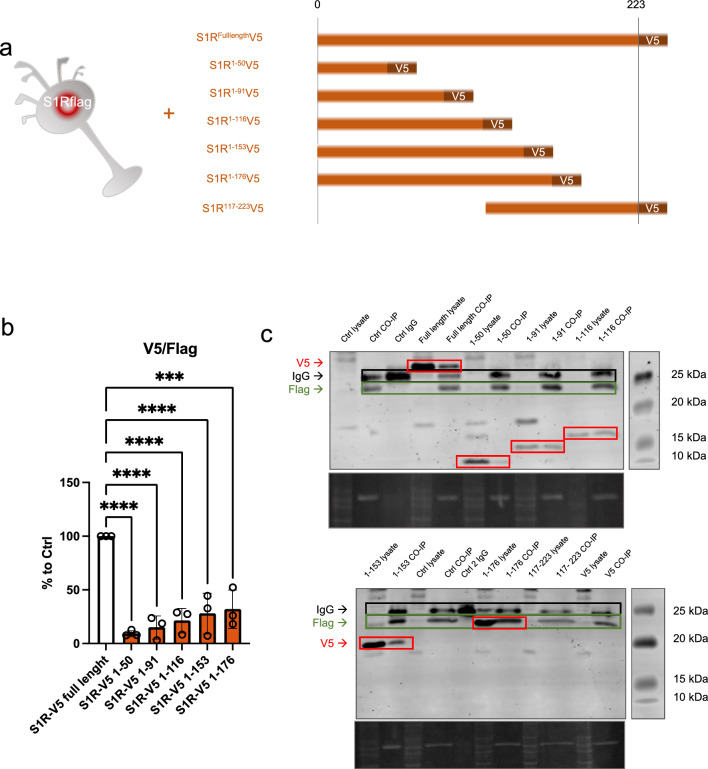
C-terminal fragment of S1R favorizes the interaction between S1Rs. N-terminal fragment seems necessary for stable expression of S1R.** a** Representation of the model used: Overexpression of S1R tagged with V5 in N2a cells expressing endogenously S1R tagged with Flag. Different truncated forms of S1R-V5 were expressed in the S1R-Flag N2a cells. **b** Bar graph represents immunoblot analysis of the S1R V5 signal intensity over the S1R-Flag intensity after immunoprecipitation of S1R-Flag. Quantity of co-immunoprecipitated S1R-V5 is highly decreased when S1R lacks the C-terminal portion. **c** Representative immunoblot showing S1R-V5, S1R-Flag, and IgG signals in co-IP samples and whole cell lysate. Significant differences were found using ordinary one-way ANOVA followed by a Tukey’s multiple comparisons test (***p < 0.001, ****p < 0.0001)

In the molecular structure of S1R, several functional amino acid (aa) groups are well identified such as the exogenous ligands binding pocket or the transmembrane domain. The parts of S1R involved in its oligomerization are however less studied. To further understand the relevance of different parts of S1R in the oligomerization process we used N2a cells expressing S1R-Flag and transfected them with different plasmids allowing the expression of truncated versions of S1R all tagged with V5 (Fig. 5a). The cellular protein contents from these different conditions were then assessed to Flag immunoprecipitation as presented in Fig. 2b. We noted that the quantity of pull-downed V5-tagged S1R following the immunoprecipitation of Flag is dependent on the C-terminus part. Interestingly we found that 176-223 aa fragment play a substantial role for the full capacity of S1R-S1R interaction. We did not see statistical differences from the different truncated S1R V5 highlighting the fact that 176-223aa seems to be the more important part of S1R involved in oligomerization. However, it should be emphasized that even if the pulldown is highly reduced without the C-terminal part of the protein, the interaction is still possible as we observe faint band of co-IPed V5 tagged S1R 1-50aa. Leading us to think that lower order oligomerization of S1R may be possible even without the C-terminus. Next, we also observed the monomers/oligomers ratio in half denaturized samples and western blot the same way we did in Fig. 1 (Supplementary Information 5a). Interestingly we observed oligomers only with the expression of the longer S1R-V5 construct (1-116, 1-153, 1-176aa), the overexpression of the shorter constructs did not present high order oligomers (1-50, 1-96aa). This suggest that the 96-116aa seem to be essential for oligomerization. We then mapped our experimental results on a crystal structure of S1R (Supplementary Information 5b, c and d). Supplementary Information 5b is an overview of a S1R receptor structure (6DJZ [46]) while 5c and d demonstrate that the 92–166 and 177–223 fragments participate in forming the oligomerization interface of S1R. Specifically we show that F191 from three monomers stack to each other, D188 and S192 establish a polar interaction network at the trimer interface and R114 from one monomer interact with the backbone of the L111 of another monomer. It must be noted that we also attempted to analyze the effect of S1R N-terminal deletion on the oligomerization process; however, we lacked adequate protein expression even in total lysate samples. One explanation for this result could be that the N-terminal part of S1R is important for the stability of mRNA and/or protein (Supplementary Information 5e).

## Discussion

The relevance of S1R as a therapeutic target is well established in various pathological context [14, 15, 47–49]. However, the mechanisms underlying S1R multifunctionality have yet to be clarified. Understanding the structural kinetics of S1R, which are known to be related to its function [27, 28], would improve our understanding of S1R and may extend the already wide range of S1R therapeutic use. In this study we first established models wherein agonists induce monomerization, even with *in-vivo* mice models, which corroborate previous results from cellular models [29–31] (Fig. 1).

**Fig. 6:**
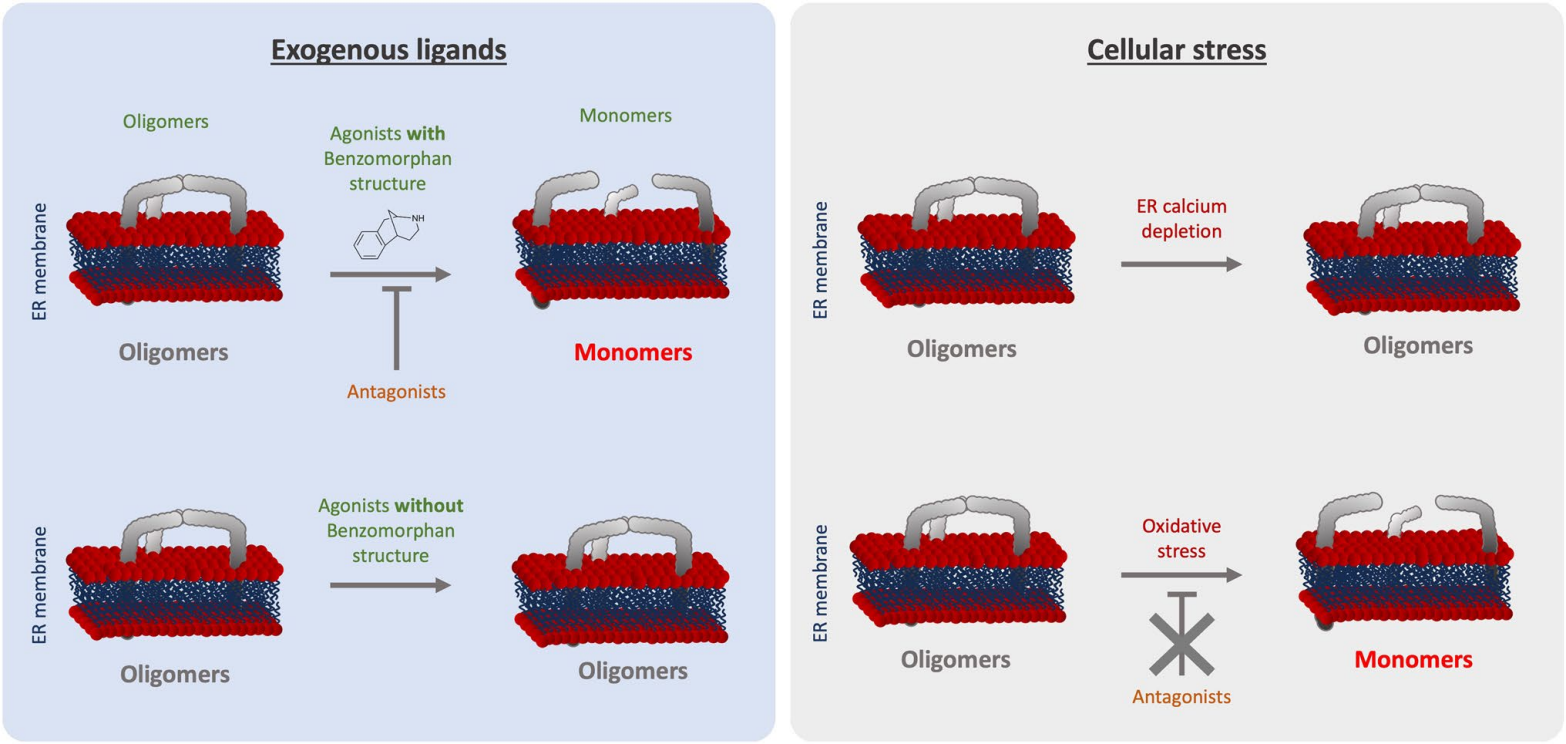
Graphical summary of the effect of S1R activators on S1R quaternary structure. (Left) schematic demonstrates the effect of exogenous ligands on S1R quaternary structure. Agonists with a benzomorphan group [( +)-Pentazocine, ( +)-SKF10047] induce monomerization which is blocked by antagonists (NE100, BD1063). Agonists without a benzomorphan group do not induce monomerization (PRE-084, Cocaine). (Right) schematic demonstrates the effect of endogenous stressors on S1R quaternary structure. ER calcium depletion does not seem to induce monomerization. Oxidative stress induces monomerization, but this is not blocked by antagonists

As we summarized in Fig. 6, we noted that ( +)-Pentazocine and ( +)-SKF10047 are able to induce monomerization of S1R. Cocaine nor PRE-084 do not induce the same effect of monomerization, despite also being considered S1R agonists. Interestingly, Wang et al*.* observes in mice model that ( +)-Pentazocine affected a wider range of phenotypes when compared to that of PRE-084 [20]. These results support the hypothesis proposed by Yano et al. that agonists, even ones with faint chemical differences, have differential effects on S1R structure and therefore function [30]. This could also explain why cocaine acts as an S1R agonist when observing specific phenotypes but does not have any effect on S1R quaternary structure [41–43] (Fig. 3). Based on these observations, we propose that S1R can be de-oligomerized by ligands with benzomorphan group [( +)-Pentazocine and SKF10047] but not by ligands lacking benzomorphan group, such as cocaine and PRE-084. As a recent publication propose to use the heterodimerization of BiP and S1R following drug application as biosensor to classify S1R ligands [50], the models we present in this article can be useful to assess new ligands.

Furthermore, these differential effects among S1R “activators” can be extended to cellular stress. In the present manuscript we showed that different cellular stress such as ER calcium depletion and oxidative stress, induced respectively with thapsigargin and H_2_O_2_, affect S1R quaternary structure differently compare to agonists (Fig. 4). Thapsigargin does not seem to alter the quaternary structure of S1R. Meanwhile H_2_O_2_ increases S1R monomerization, this is not blocked by S1R antagonist. The transport or diffusion of S1R ligands through the plasma membrane is not well understood. Thus, lead us to hypothesize that applying S1R antagonist at different points could potentially affect the effect of H_2_O_2_. Our findings support adopting a novel perspective on S1R activation as a nuanced phenomenon dependent on the type of stimulus. Nevertheless, the molecular mechanism beyond the H_2_O_2_ trigger of S1R remains unclear. The main questions that remain are: Does H_2_O_2_ acts directly or indirectly on S1R via its effect on endogenous ligands? Do other reactive oxygen species act the same way on S1R quaternary structure? Further *in-vivo* experiments, independent of the S1R context could help to answer these questions and shed the light on this interaction.

Another area for future research on S1R structure is the difference between dimer organization and trimer (or higher) organization. It would be interesting to observe what part of S1R is involved in homomerization, and also what differences, if any, exist between the dimerization and trimerization processes. We believe that determining the different functions among different orders of oligomerization will clarify the therapeutic potential of S1R. In fact, the S1R E102Q mutation is responsible for the development of a genetic form of ALS [51] and interestingly, Abramyan et al*.* observe that this mutation also destabilizes higher order S1R oligomers [52]. These findings further strengthen the relationship of S1R oligomerization with its function. Of note, it seems that the S1R E102Q mutation and exogenous ligand activation both cause S1R monomerization. Furthermore, Sawyer et al*.* observe that S1R mutations of amino acids contained in the domains, highlighted in Fig. 5, decrease S1R oligomerization and impact rough ER shaping [28]. More experiments are needed to determine the exact consequences of S1R structural changes on S1R’s effects on cell survival. For example, different orders of oligomerization may differentially affect the ability of S1R to interact with partners (e.g., BiP, Ip3R3, IRE1) or to translocate along the ER membrane. This information would allow researchers to strategically test S1R ligands that affect oligomerization according to the pathological context and therapeutic need.

In this report we demonstrate that S1R quaternary structural changes depend on the nature of S1R activation. More specifically, we discovered that well-known S1R agonists promote different quaternary structural changes depending on the presence or absence of benzomorphan group, suggesting for the first time that S1R agonists have sub-groups. The existence of S1R sub-groups could refine the selection of S1R agonists for therapeutic use, for more specific targeting of pathological contexts.

### Supplementary Information

Below is the link to the electronic supplementary material.

Supplementary file1 (DOCX 458 KB)

## Data Availability

The datasets analyzed during the current study are available from the corresponding author on reasonable request.

## Abbreviations

ALS: Amyotrophic lateral sclerosis
BiP: Binding immunoglobulin protein
ER: Endoplasmic reticulum
GFP: Green fluorescent protein
Ip3R3: Inositol 1,4,5-trisphosphate receptor type 3
IRE-1: Inositol-requiring enzyme 1
KO: Knock-out
MAM: Mitochondria associated endoplasmic reticulum-membrane
S1R: Sigma-1 receptor
UPR: Unfolded protein response
Wt: Wild-type
