# Supplementary material for: Benzomorphan and non-benzomorphan agonists differentially alter sigma-1 receptor quaternary structure, as does types of cellular stress
Source: Cell Mol Life Sci. 2024 Jan 9;81(1):14. doi: 10.1007/s00018-023-05023-z (PMC10774196; doi:10.1007/s00018-023-05023-z)

**Supplementary Information 1**

**Effect of temperature during SDS-PAGE samples analyzing on S1R western blot signal.** **(a)** Immunoblot showing the effect of thermal denaturation on S1R oligomerization. Monomers are observed around 24kDa and present in both boiled and non-boiled conditions. Oligomers (75kDa and 150 kDa) are only present in non-boiled condition. **(b)** Immunoblot showing results from S1R oligomers and monomers in three independent experiments between S1R KO N2a cells and S1R KO N2a cells overexpressing S1R-YFP or only YFP. (c) Concentration of endogenous monomers and oligomers in control conditions of N2a cells and mice livers. Exp A represents signals coming from control samples presented in Figure 1d. Exp B represents signals coming from control samples presented in Figure 1f. Mice liver extracts B represents signals coming from control samples presented in Figure 1j. (d) Ratio monomers/oligomers between *in-vitro* and *in-vivo* experiments. Significant differences were found using t-test or ordinary one-way ANOVA followed by a Tukey's multiple comparisons test (* p<0.05)

**Supplementary Information 2**

**Flag-tagging of endogenous in N2a cells (a)** Immunoblot showing protein contents from Wt N2a, S1R-Flag N2a cells (clone #8, #13), and S1R KO N2a cells (KO). Upper picture shows Flag labelled membrane, middle picture shows S1R signal, and the lower picture shows the merging of both signals. Clone #13 was selected for the rest of the experiments. **(b)** Sequencing from #13 showing the Flag sequence followed by the stop codon of S1R sequence. **(c)** Immunoblot showing S1R immunoprecipitation with anti-Flag antibody pulldown. **(d)** Immunoblot showing S1R co-immunoprecipitation with IP3 using anti Flag antibody pulldown.

**Supplementary Information 3**

**Effect of Cocaine and BD1063 on quaternary structure of S1R. (a)** Representation of the model used: Overexpression of S1R tagged with YFP in S1R KO N2a cells. **(b left)** Bar graph represents immunoblot analysis of the S1R ratio mono/oligo in control condition or with incubation of cocaine for 30 min at different concentration (1 to 100 µM). None of the dosages affect S1R quaternary structure. **(b right)** Representative immunoblot showing S1R-YFP oligomers and monomers after incubation with cocaine. **(c left)** Bar graph represents immunoblot analysis of the S1R ratio mono/oligo in control condition or with incubation of cocaine for 24 h at different concentration (1 to 100 µM). None of the dosages affect S1R quaternary structure. **(c right)** Representative immunoblot showing S1R-YFP oligomers and monomers after incubation with cocaine. **(d left)** Bar graph represents immunoblot analysis of the S1R ratio mono/oligo in control condition or with incubation of (+)-Pentazocine (10 µM) for 30 min with or without the pre-incubation for 5 min and co-incubation of cocaine at different concentrations (1 to 100 µM). None of the cocaine dosages attenuate the effect of (+)-Pentazocine on S1R quaternary structure. **(d right)** Representative immunoblot showing S1R-YFP oligomers and monomers after incubation with (+)-Pentazocine with or without the pre-incubation and co-incubation of cocaine. **(e)** Representation of the model used: Endogenous S1R in Wt N2a cells. **(f left)** Histogram showing the dose-dependent effect of SKF-10047 (from 0.03 to 300 µM). **(f right)** Representative immunoblot showing S1R oligomers and monomers after incubation with SKF-10047. **(g left)** Bar graph represents immunoblot showing S1R oligomers and monomers after incubation with BD1063. **(g right)** Representative immunoblot showing S1R oligomers and monomers after incubation with BD1063. Significant differences were found using ordinary one-way ANOVA followed by a Tukey's multiple comparisons test.

**Supplementary Information 4**

**Effect of Thapsigargin and H_2_O_2_ on protein concentration of cell extract. (a)** Protein concentration measurement for each independent cell culture treated with a different concentration of thapsigargin. No significant differences are observed among conditions. **(b)** Protein concentration measurement for each independent cell culture treated with a different concentration of H_2_O_2_. No significant differences are observed among conditions using ordinary one-way ANOVA followed by a Tukey's multiple comparisons test.

**Supplementary Information 5**

**C-terminal truncated S1R-V5 (1-96) is unable to oligomerize and N-terminal truncated mutant (117-223) shows decreased mRNA concentration.** **(a)** Immunoblot showing the effect of thermal denaturation on S1R V5 constructions with different lengths: 1-50aa, 1-96aa, 1-116aa, 1-153aa, 1-176aa, 117-223aa. Non-boiled samples allow observation of high order oligomers compared to that of boiled samples, when using longer constructs of S1R (1-116, 1-153, 1-176). The shorter constructs are unable to form high order oligomers (1-50, 1-96). S1R 117-223 V5 is present in a very low concentration compared to the other S1R constructions. **(b)** Overview of a S1R structure (6DJZ) with the segments 92-166 and 177-233 from monomers A, B, C colored differently. **(c)** Zoom-in view of the 177-233 region, enclosed by the dotted boxes in d and showing the representative interactions of these two segments that contribute to form the trimer interface. Specifically demonstrates that F191 from three monomers stack to each other, while D188 and S192 establish a polar interaction network at the trimer interface. **(d)** Zoom-in view of the 92-166 region, enclosed by the dotted boxes in d and showing R114 from one monomer interacting with the backbone of the L111 of another monomer. **(e left)** Bar graph representing qPCR results of S1R transcript with amplification of C-terminal sequence in S1R KO N2a cells (ctrl) and S1R KO N2a cells overexpressing S1R V5^fulllength^, S1R^117-223^ or S1R^1-50^. **(e right)** Table with qPCR values normalized to ctrl condition.


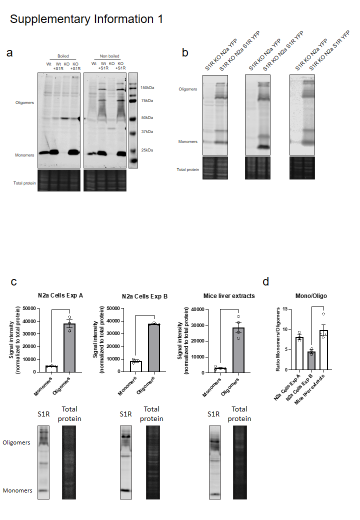


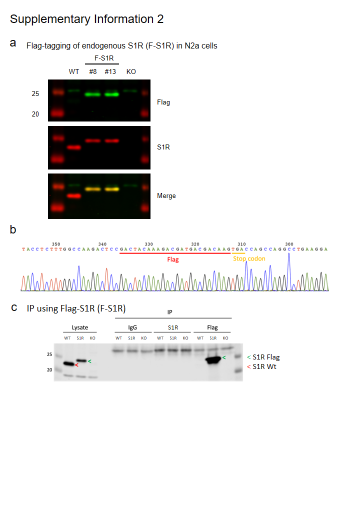


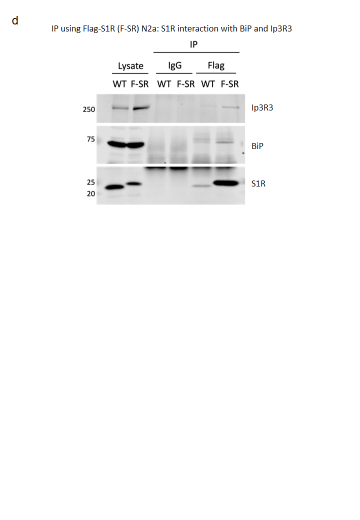

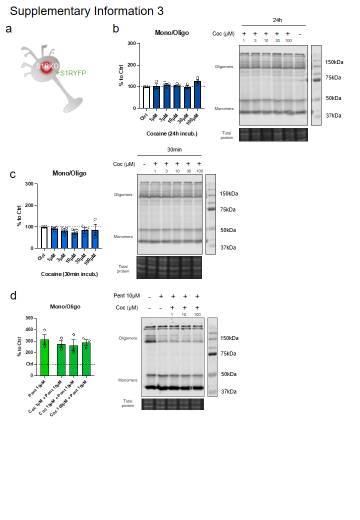

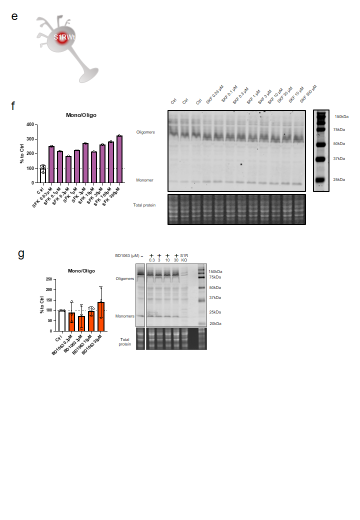

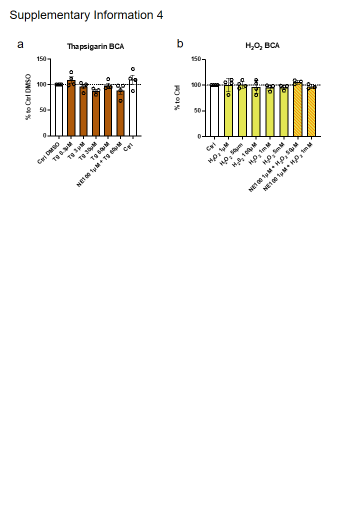

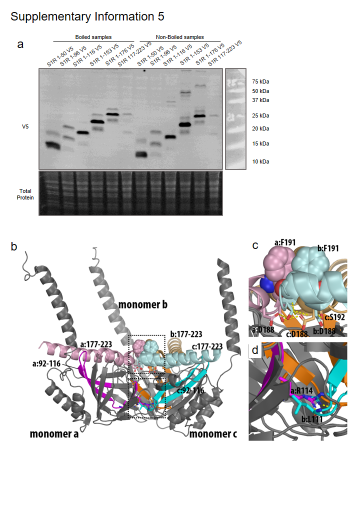

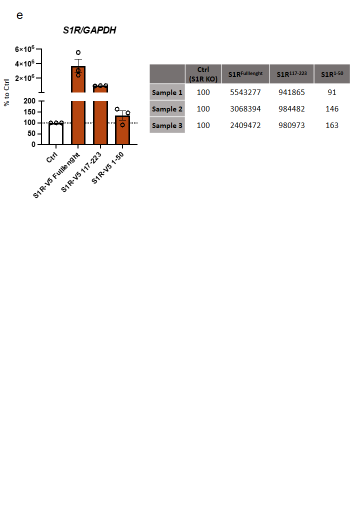

Supplement: Supplementary file 1 — Supplementary file1 (DOCX 458 KB) [file 18_2023_5023_MOESM1_ESM.docx]
